# *Marantodes pumilum* var. *alata* Enhances Fracture Healing Through Gene Regulation in a Postmenopausal Rat Model

**DOI:** 10.3390/ph18050736

**Published:** 2025-05-16

**Authors:** Tijjani Rabiu Giaze, Norazlina Mohamed, Syed Alhafiz Syed Hashim, Ahmad Nazrun Shuid, Ima Nirwana Soelaiman, Norliza Muhammad, Fadhlullah Zuhair Jafar Sidik, Jamia Azdina Jamal

**Affiliations:** 1 Department of Pharmacology, Faculty of Medicine, Universiti Kebangsaan Malaysia, Cheras, Kuala Lumpur 56000, Selangor, Malaysia; rtgiaze1143@gmail.com (T.R.G.); syedalhafiz@ukm.edu.my (S.A.S.H.); imasoel@hctm.ukm.edu.my (I.N.S.); norliza_ssp@hctm.ukm.edu.my (N.M.); 2 Department of Pharmacology and Toxicology, Usmanu Danfodiyo University, Sokoto 2346, Nigeria; 3 Institute of Pharmaceutical Science, King’s College London, Franklin-Wilkins Building, 150 Stamford Street, London SE1 9NH, UK; 4 Department of Pharmacology, Faculty of Medicine, Universiti Teknologi Mara, Sungai Buloh 47000, Selangor, Malaysia; anazrun@yahoo.com; 5 Faculty of Pharmacy, Universiti Kebangsaan Malaysia, Cheras, Kuala Lumpur 56000, Selangor, Malaysia; jffz@ukm.edu.my (F.Z.J.S.); jamia@ukm.edu.my (J.A.J.)

**Keywords:** fracture, gene expression, *Marantodes pumilum*, osteoporosis, ovariectomized rats

## Abstract

**Background:** *Marantodes pumilum* var. *alata* (*MPva*) has been reported to promote fracture repair. This study investigates the role of *MPva* leaf extract on biochemical markers and bone-repair genes in a postmenopausal rat model to understand its fracture-healing properties. **Methods**: Thirty female Sprague Dawley rats were grouped into sham-operated (Sham), ovariectomized control (OVXC), estrogen treatment (ERT), and plant treatment (MPv20 and MPv100) groups. After ovariectomy, the right tibiae of rats were fractured. The ERT group was treated with 64.5 μg/kg/day of estrogen, while the MPv20 and MPv100 groups received 20 and 100 mg/kg/day doses of *MPva* leaf extract, respectively, for 8 weeks. Sham and OVXC acted as untreated controls. Blood samples collected before and after treatment were assayed for pro-inflammatory cytokines (IL-6 and TNF-α), while bone samples were assayed for bone-turnover markers: osteocalcin and pyridinoline, oxidative-status markers (GPx, SOD, and MDA), and bone-repair genes (*Bglap*, *Spp1*, *Dkk1*, *Igf1*, *Tnfsf11*, and *Fgf23*). **Results**: IL-6, GPx, and SOD levels were significantly increased in both MPv groups (*p* < 0.05). *IGF1* was significantly upregulated in both MPv groups, while *Tnfsf11* was downregulated in the MPv20 group (*p* < 0.05). **Conclusions**: *MPva* leaf extract may promote bone repair by stimulating pro-inflammatory and antioxidant responses, which are associated with its regulation of *Igf1* and *Tnfsf11 genes*.

## 1. Introduction

Bone fractures are among the most common and life-threatening complications of osteoporosis [1]. With increasing life expectancy and an aging population, the incidence of osteoporotic fractures is projected to surge significantly. Globally, these fractures substantially burden healthcare systems, particularly in regions like Asia. In Asia, the number of hip fractures is expected to rise from 1,124,060 in 2018 to 2,563,488 in 2050, resulting in a 1.59-fold increase in direct cost [2].

Fracture healing is a complex, sequential process involving three stages: reactive, reparative, and remodeling [3]. The reactive or inflammatory phase of fracture healing occurs within the first two weeks after fracture, followed by the reparative stage (callus formation), which continues until the sixth week after fracture [4]. The remodeling phase will then occur, and it typically extends for weeks or months after fracture, restoring the injured bone to its pre-fracture anatomy and physiological function. Throughout these stages, numerous cellular and molecular pathways interact, triggering a cascade of events involving local and systemic regulatory factors, such as bone morphogenetic proteins (BMPs), growth factors (TGF-β, PDGFs, and FGFs), angiogenic factors, and metalloproteinases. These factors communicate with certain cell types, like mesenchymal stem cells (MSCs) and osteoprogenitor cells [5], to regenerate bone. However, this process is highly sensitive to both local and systemic influences, including hormonal imbalances and inflammatory states.

Fracture-repair responses vary significantly between individuals, with estrogen deficiency strongly associated with delayed fracture healing [6]. Estrogen regulates vascular endothelial growth factor (VEGF) production and promotes osteoprotegerin (OPG) secretion to prevent osteoclast maturation, while also maintaining Bcl-2 levels to support osteoblast survival and reduce apoptosis [7]. Postmenopausal osteoporosis, characterized by reduced estrogen levels, not only weakens bone structure, but also disrupts the natural healing process. Altered inflammatory responses further compound these challenges [8], contributing to impaired fracture repair in postmenopausal osteoporosis [9,10]. Inflammation plays a crucial role in the initial stages of fracture healing [11,12], but excessive or prolonged inflammatory responses can delay tissue regeneration and hinder recovery, exacerbating healing complications in estrogen-deficient individuals.

While conventional antiresorptive drugs, such as bisphosphonates, have been shown to improve bone mineral density (BMD) and reduce the risk of fractures [13,14], their role in enhancing fracture repair remains controversial [15,16]. Concerns about the adverse effects of commercially available antiresorptive agents [17] have spurred interest in developing alternative or complementary remedies for managing osteoporosis and related fractures [18,19]. Effective therapies must address the multifactorial nature of fracture healing, targeting key pathways such as hormonal regulation, oxidative stress, and inflammation. By adopting such a comprehensive approach, new solutions could significantly improve bone health and recovery outcomes in osteoporotic conditions.

One such potential remedy is *Marantodes pumilum* var. *alata* (*MPva*). This plant has demonstrated beneficial effects on postmenopausal conditions linked to estrogen deficiency [20,21]. Previous studies have shown that *MPva* preserves bone density, maintains microarchitecture, and prevents loss of mechanical strength in ovariectomized rats [22,23]. In 2018, we reported that its leaf extract promotes fracture healing in postmenopausal rats by increasing bone callus production and strength [24]. Phytochemical analyses revealed the presence of bioactive compounds such as phytoestrogens, β-carotene, quercetin, myricetin, kaempferol, catechin, gallic acid, ellagic acid, and vanillic acid, which may underlie these effects [25,26].

Despite these promising findings, further scientific evidence is needed to substantiate *MPva*’s fracture-repair properties in osteoporotic conditions. Understanding its biochemical and genetic mechanisms of action could provide a foundation for its clinical application. This study, therefore, aims to further investigate the fracture-healing effects of aqueous leaf extract of *MPva* by evaluating its influence on bone-turnover markers, oxidative status, and pro-inflammatory cytokines, as well as fracture-repair genes, in a postmenopausal rat model.

## 2. Results

### 2.1. Body Weight

At the end of the first week of treatment, a significant increase in the body weight of rats in the OVXC group was observed when compared with the Sham group (*p* < 0.05). Like the OVXC group, rats treated with estrogen also recorded significantly higher body weights compared to the Sham group (*p* < 0.05). Similarly, rats treated with 20 and 100 mg/kg doses of the leaf extract recorded significantly higher body weights when compared to the Sham group (*p* < 0.05) (Table 1).

### 2.2. Pro-Inflammatory Cytokines

**Figure 1:**
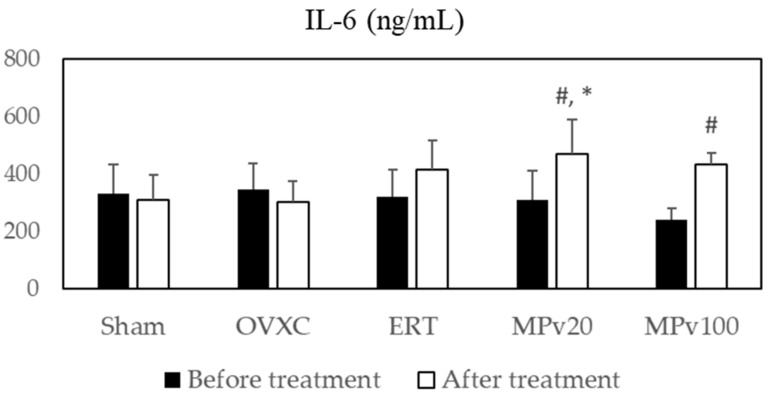
Serum levels of IL-6 in ovariectomized rats treated with *Marantodes pumilum* var. *alata* leaf extract. Data presented as mean ± SEM (*p* < 0.05, ANOVA). # indicates significant difference compared to pre-treatment levels within same group; * indicates significant difference compared to post-treatment levels in OVXC group. Sham: sham-operated; OVXC: ovariectomized control; ERT: estrogen treatment 64.5 μg/kg/day; MPv20: 20 mg/kg leaf extract of *Marantodes pumilum*; and MPv100: 100 mg/kg leaf extract of *Marantodes pumilum*.

**Figure 2:**
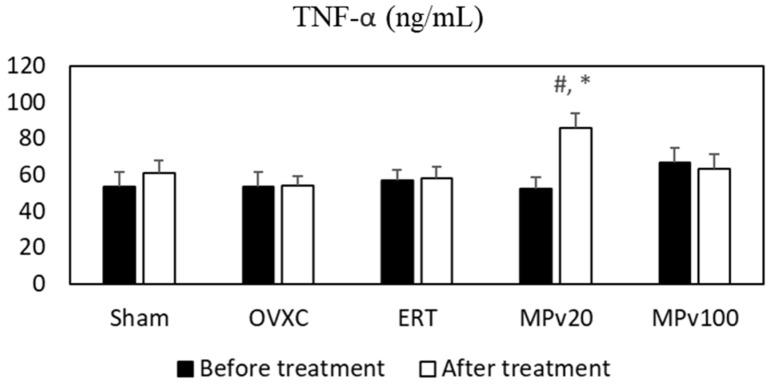
Serum levels of TNF-α in ovariectomized rats treated with *Marantodes pumilum* var. *alata* leaf extract. Data presented as mean ± SEM (*p* < 0.05, ANOVA). # indicates significant difference compared to pre-treatment levels within same group; * indicates significant difference compared to post-treatment levels in OVXC group. Sham: sham-operated; OVXC: ovariectomized control; ERT: estrogen treatment 64.5 μg/kg/day; MPv20: 20 mg/kg leaf extract of *Marantodes pumilum*; and MPv100: 100 mg/kg leaf extract of *Marantodes pumilum*.

No significant differences in the levels of IL-6 or TNF-α were observed between the OVXC and Sham groups (Figure 1 and Figure 2).

Treatment with *MPva* significantly elevated serum IL-6 levels in both the MPv20 and MPv100 groups compared to their respective baseline levels (*p* < 0.05) (Figure 1). Interestingly, the post-treatment IL-6 concentration in the MPv20 group was significantly higher than that in the OVXC group (*p* < 0.05) (Figure 1).

Serum TNF-α levels were significantly increased after treatment relative to baseline (*p* < 0.05) in the MPv20 group (Figure 2). Furthermore, post-treatment TNF-α levels in the MPv20 group were significantly higher than those in the OVXC group (*p* < 0.05) (Figure 2).

### 2.3. Oxidative Status Markers

The OVXC group showed a lower bone level of the GPx enzyme than the Sham group, but the difference did not reach the significance level (Table 2). However, the OVXC group had a significantly lower GPx enzyme level compared to the leaf extract and estrogen treatment groups (MPv20, MPv100, and ERT). Additionally, the bone levels of SOD were found to be significantly lower in the OVXC group when compared with the Sham group (*p* < 0.05). Similarly to the Sham group, significantly higher bone levels of SOD were also seen in both the MPv20 and MPv100 treatment groups compared to the OVXC group (*p* < 0.05). There was no significant difference in MDA values among the different groups.

### 2.4. Bone-Turnover Markers

**Figure 3:**
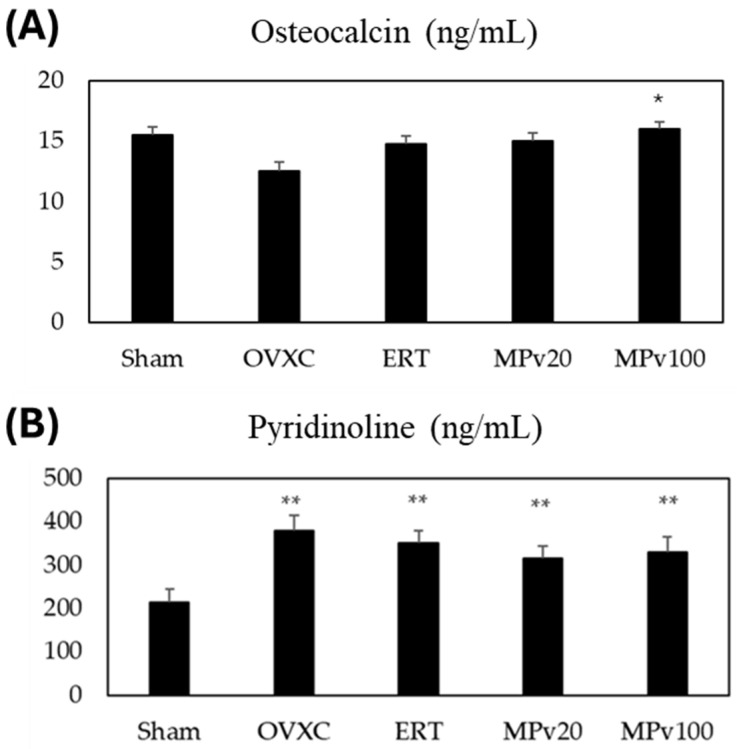
Levels of bone-turnover markers (**A**) osteocalcin and (**B**) pyridinoline in fractured tibia of ovariectomized rats treated with *Marantodes pumilum* var. *alata* leaf extract. Data presented as mean ± SEM (*p* < 0.05, ANOVA). * indicates significant difference compared to OVXC group; ** indicates significant difference compared to Sham group. Sham: sham-operated; OVXC: ovariectomized control; ERT: estrogen treatment 64.5 μg/kg/day; MPv20: 20 mg/kg leaf extract of *Marantodes pumilum*; and MPv100: 100 mg/kg leaf extract of *Marantodes pumilum*.

No significant difference in the bone levels of osteocalcin were seen between the OVXC group and the Sham group. The bone level of osteocalcin was only found to be significantly higher (*p* < 0.05) in the MPv100 group compared to the OVXC group (Figure 3A). The pyridinoline levels in the OVXC and treatment groups were significantly higher (*p* < 0.05) compared to that in the Sham group. However, the levels of pyridinoline in the estrogen, MPv20, and MPv 100 treatment groups were not different to that in the OVXC group (Figure 3B).

### 2.5. QuantiGene Plex Assay

**Figure 4:**
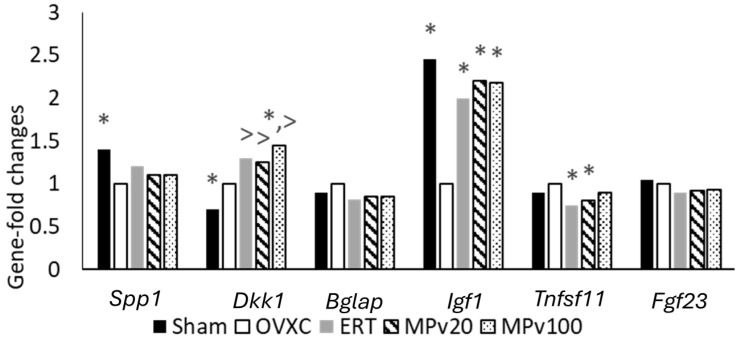
Mean fold changes in normalized bone-repair genes of ovariectomized rats treated with *Marantodes pumilum* var. *alata* leaf extract. * indicates significant difference compared to OVXC group. > indicates significant difference compared to Sham group. Sham: sham-operated; OVXC: ovariectomized control; ERT: estrogen treatment 64.5 μg/kg/day; MPv20: 20 mg/kg leaf extract of *Marantodes pumilum*; and MPv100: 100 mg/kg leaf extract of *Marantodes pumilum*.

Differences in the fold changes in expressed genes, as shown in Table 3, were recorded across treatment groups in comparison to the OVXC group after normalizing the median fluorescence intensities (MFIs) with the housekeeping genes (HKGs). Further analysis, utilizing the geometric mean of the housekeeping genes, revealed significantly higher (*p* < 0.05) fold changes in *Dkk1* in the MPv100 group compared to the OVXC group (Figure 4). In addition, similarly to the ERT and Sham groups, significantly higher (*p* < 0.05) fold changes in Igf1 were recorded in both the MPv20 and MPv100 groups compared to the OVXC group, while similarly to ERT group, significantly lower (*p* < 0.05) fold changes in RANKL (*Tnfsf11*) were recorded in the MPv20 group compared to the OVXC group.

## 3. Discussion

Following a fracture, pro-inflammatory cytokines such as IL-6 [27] and TNF-α [28] play a crucial role in initiating injury-repair processes. These cytokines are immediately secreted at the injury site by macrophages, inflammatory cells, and mesenchymal-origin cells, enhancing extracellular matrix synthesis and stimulating angiogenesis [29]. In this study, treatment with a 20 mg/kg/day dose of *MPva* leaf extract significantly elevated the serum levels of IL-6 and TNF-α in rats (*p* < 0.05). Low-dose TNF-α administration has been shown to promote neutrophil and monocyte recruitment, enhancing fracture healing [30]. TNF-α levels are elevated during different phases of fracture healing, including the early inflammation phase and the later remodeling phase [31]. Similarly, IL-6 influences fracture healing during the early and repair phases [32]. Typically, pro-inflammatory cytokine secretion peaks within the first 24 h post injury and rapidly declines to near-zero levels by day three [33]. Due to bone remodeling activities seen in secondary bone formation, a second rise in the levels of IL-6 and TNF-α has been reported [34]. Here, a 100 mg/kg/day dose of *MPva* also elevated serum IL-6 levels (*p* < 0.05). The elevated cytokine levels observed in this study indicate that the aqueous *MPva* leaf extract, at lower doses, promotes the release of pro-inflammatory cytokines. This release may trigger inflammatory responses that are beneficial for initiating fracture repair, supporting bone remodeling during secondary bone formation, and facilitating mineralized cartilage resorption at the endochondral phase. This release plays a key role in initiating fracture repair, supporting bone remodeling during later stages, and facilitating the resorption of mineralized cartilage in the endochondral phase. Although the IL-6 and TNF-α levels in this study exceeded typical physiological limits, these elevations may represent an increased but compensatory response to fracture and estrogen deficiency, rather than overtly pathological inflammation. This suggests that, under certain conditions, heightened cytokine activity may still contribute positively to healing. Nevertheless, maintaining a balanced cytokine response remains critical, as excessive or prolonged elevations can risk chronic inflammation or impaired recovery. The effects of *MPva* may not rely solely on cytokine modulation, but may also involve alternative mechanisms, such as IGF1 upregulation, antioxidant support, and modulation of bone-related genes, as observed elsewhere in our study.

Estrogen deficiency has been shown to increase reactive oxygen species levels [35] and reduce antioxidant enzyme gene expression, thereby delaying fracture healing [36]. The total antioxidant capacity decreases during the first two weeks of fracture healing [37], which aligns with reductions in antioxidant enzymes reported in ovariectomized, fractured animals [38]. In this study, the significant reduction in GPx enzyme activity observed in estrogen-deficient controls was reversed by the *MPva* leaf extract at both 20 and 100 mg/kg/day doses (*p* < 0.05), mimicking estrogen treatment. Similarly, *MPva* treatment prevented SOD enzyme loss in untreated estrogen-deficient controls (*p* < 0.05). These findings suggest that *MPva* leaf extract may support fracture healing by providing antioxidant protection to fractured tibiae. Similar antioxidant effects have also been observed with other medicinal plants, like *Urtica urens* extract, which improved oxidative stress markers and supported bone remodeling in rats exposed to toxic injury [39]. However, despite the upregulation of GPx and SOD, MDA levels remained unchanged, which have may been due to *MPva*’s antioxidant effect mitigating oxidative damage, preventing further increases in MDA and maintaining levels at baseline during fracture healing.

Bone-turnover markers in serum or urine correlate with bone-turnover activities, and are commonly assessed during fracture risk evaluation and antiresorptive drug therapy [40]. In the current study, higher doses of *MPva* leaf extract increased serum osteocalcin levels, indicating enhanced bone formation activities, an absent effect in both estrogen-treated and healthy controls. Serum osteocalcin is considered a potential risk factor for predicting fracture susceptibility in postmenopausal women [41]. The level of the bone resorption marker PYD was elevated in the OVX rats compared to controls, reflecting increased bone resorption activity. Increased bone resorption is known to play a role in the non-union of fractured bone [42]. Groups treated with *MPva* also showed a high PYD levels compared to control rats. This suggests that *MPva* does not affect bone resorption in the context of promoting fracture healing. A previous study on *Labisia pumila* extract supports these findings, showing effects on osteoblastic bone formation markers, but not on bone resorption markers, in ovariectomized rats [43].

QuantiGene 2.0 assays revealed variations in the expression of genes involved in fracture repair. Treatment with both 20 and 100 mg/kg/day doses of *MPva* leaf extract, similarly to estrogen treatment, induced a significant increase in fold changes in the *IGF1* gene compared to estrogen-deficient controls (*p* < 0.05). IGF1, a member of the insulin-like growth factor (IGF) family, has been reported to promote bone healing [44,45]. Its mechanism in bone regeneration may be attributed to its ability to induce osteogenic differentiation [46]. Thus, the upregulation of *Igf1* gene expression following treatment with the leaf extract of *MPva* may be partly responsible for the fracture-healing property of *MPva*.

*TNFSF11*, also known as RANKL, plays a role in osteoclastogenesis, thus leading to loss of mineralization [47]. RANKL has been shown to be negatively associated with bone mineral density in postmenopausal women [48]. During fracture healing, a higher level of RANKL and OPG was observed at baseline and at week 4 after injury. The level declined after 4 weeks [49]. In an animal study, the RANKL level remained elevated even at 12 weeks after fracture, which delayed healing [50]. Treatments with a 20 mg/kg/day dose of *MPva* leaf extract and estrogen were able to prevent the increase (*p* < 0.05) in fold changes in the *TNFSF11* gene seen in the estrogen-deficient control. In view of the role of the *TNFSF11* gene in fracture repair, this outcome signifies that treatment with *MPva* leaf extract is capable of promoting fracture repair by modulation of RANKL. RANKL has been reported to be responsible for the non-union of fracture due to its bone-resorptive role during fracture repair [42].

Additional QuantiGene assay outcomes also revealed an increase in fold changes in the *Dkk1* gene in the untreated estrogen-deficient control compared to the healthy control. DKK1, a potent Wnt antagonist, is known to cause blockade of Wnt/β-catenin signaling in osteoblasts by binding to Wnt co-receptor lipoprotein-related protein-5 (LRP5), thus inhibiting its development and function [51]. DKK1 has been found to be increased in postmenopausal women, which correlates with the severity of osteoporosis [52]. DKK1 has also been shown to cause bone loss by decreasing the expression of osteogenic markers [53]. Diminished DKK1 levels resulting from a lack of a functional *Dkk1* allele result in alterations in bone development and patterning and an increase in bone mass [54].

Treatment with *MPva* leaf extract and estrogen, rather than preventing the rise in the *Dkk1* gene seen in the untreated estrogen-deficient control, caused an increase in fold changes in the *Dkk1* gene. The levels in rats treated with a 100 mg/kg/day dose of *MPva* leaf extract were found to be significantly higher compared to those in the untreated estrogen-deficient controls (*p* < 0.05). Despite this, fracture healing was enhanced, likely due to the upregulation of IGF1, a key promoter of osteoblast proliferation and bone formation. The concurrent elevation of *Igf1* and *Dkk1* suggests that *MPva*-induced healing may occur independently of the DKK1/Wnt signaling axis. Rather than reflecting a direct interaction, the upregulation of DKK1 may be unrelated to the observed bone repair, which appears to be primarily driven by IGF1 or other osteogenic pathways.

In the current study, the body weight of the animals was also measured. Significant body weight gain was observed in the OVXC group compared to the Sham group. Similar observations were noted for the other treatment groups. In a previous study, it was found that rats treated with a 50 mg/kg dose of *MPva* (*Labisia pumila*) showed less weight gain compared to the ovariectomized control [55]. The variation in body weight findings seen can be attributed to differences in the nature of the plant extract used, as the whole plant was used in the previous study, while leaf extract was utilized in this study. However, food intake was not measured in this study; thus, the detailed effect of leaf extract on body weight could not be ascertained. In our previous study, we characterized the phytochemical composition of *Marantodes pumilum* var. *alata* (*MPva*) using LC-MS/MS analysis and identified gallic acid and ellagic acid as its major bioactive compounds, as shown in the chromatograms in Figure 1a,b and summarized in Table 2 of the paper [23]. The leaves contained a higher concentration of gallic acid (5.540 µg/mL) compared to the roots (2.240 µg/mL), while the roots had a greater amount of ellagic acid (0.115 µg/mL), which was double the amount found in the leaves. Other flavonoids, including caffeic acid, myricetin, apigenin, kaempferol, and quercetin, were below the detection limits [23]. Given the established osteoprotective properties of gallic and ellagic acids, their presence in *MPva* extracts may contribute to the bone-healing effects observed in this study.

Overall, the findings in this study support claims of the bone-healing property of *MPva* leaves in an ovariectomized rat model. The potency of the effects of the *MPva* leaf extract on fracture-repair activities was found to be similar to that seen for estrogen treatment. Although treatment with both doses of *MPva* leaf extract showed similar research outcomes in this study, a higher dose (100 mg/kg) of *MPva* leaf extract showed more consistency. Gallic and ellagic acid contained in the aqueous extracts of *MPva* leaves, which have been shown to exert osteoprotective properties [23], may also be responsible for these fracture-repair activities. Although the study design did not encompass all phases of fracture repair and the results are indirectly extrapolated to humans, the findings offer valuable insights into the potential mechanisms of action of *MPva*. These results could serve as a foundation for future studies exploring the clinical applications of *MPva* leaf extract in bone healing. When translating these findings to clinical practice, the equivalent doses of 20 mg/kg and 100 mg/kg in rats would correspond to approximately 300 mg and 1000 mg for a 60 kg human, respectively, based on body surface area calculations [56].

This study provides meaningful insights into the potential of *MPva* leaf extract in promoting fracture healing. However, several limitations should be considered for future research. The current study focused primarily on biochemical and gene expression analysis. However, earlier studies from our group have already demonstrated the mechanical strength of fractured femora through three-point bending tests, alongside radiographic evidence of enhanced bone microarchitecture [24] and histological confirmation of improved bone structure [57]. These prior assessments provide structural and functional confirmation of the osteoprotective effects of *MPva* leaf extract. Future research could expand on these findings by incorporating long-term studies to evaluate the continued efficacy and safety of *MPva* in bone healing, as well as clinical trials to determine its therapeutic potential in humans. Although gene expression was analyzed here, the study did not include protein-level validation, which is crucial for confirming the translation of genetic changes into functional proteins involved in fracture repair. The roles of IGF1 and DKK1 in bone remodeling also warrant further investigation, particularly through in vitro studies, to better understand their combined or opposing effects on osteoblast and osteoclast function. Addressing these limitations in future studies will enhance the understanding of *MPva*’s mechanisms of action and support its potential application in clinical bone healing.

## 4. Materials and Methods

### 4.1. Plant Extract Preparation

The leaves of *Marantodes pumilum* var. *alata* (commonly known as ‘Kacip Fatimah’ ‘Seluso Fatimah’, ‘Kacit Fatimah’, and ‘Rumput Palis’) were collected on 16 February 2016 from a cultivated site (293Q+6X Simpang Empat) in Kedah, Malaysia. The plant was authenticated by Professor Emeritus Dr. Abdul Latiff Mohamad of the Faculty of Science and Technology, Universiti Kebangsaan Malaysia (UKM). A voucher specimen (No: UKM-HF131) was prepared and deposited at the UKM Herbarium. The collected leaves were garbled, then dried to a stable weight in the shade for 14 days. The dried leaves were ground using a rotary grinder. Following a previously described method [58], 1000 g of leaves underwent extraction in 3 L of distilled water using a reflux extraction method at 60 °C for 2 h. The resulting extracts were freeze-dried to obtain a dry extract, which was weighed and stored at −20 °C for experimental use.

### 4.2. Experimental Animals

All experimental procedures were approved by the UKM Animal Ethics Committee (Approval No: FP/FAR/2016/NORAZLINA/28-JAN./720), dated 28 January 2016, covering the period from January 2016 to December 2017. Thirty (30) female Sprague Dawley rats obtained from the UKM animal laboratory unit (weighing 300 ± 5 g) were first acclimatized to the laboratory environment. They were housed under room temperature, natural humidity, and a day–night cycle, with six rats per cage (*n* = 6) in plastic cages furnished with wood shavings. The animals were allowed free access to potable water and a standard diet containing 0.97% calcium, 0.85% phosphorus, and 1.05 IU/g vitamin D3 (Gold Coin, Rawang, Selangor, Malaysia). All rats, except for those in the sham-operated group, were ovariectomized under anesthesia (Ketamine: Xylazine, 8:1) as previously described [59]. After ovariectomy, the rats were left untreated for eight weeks to develop osteoporotic condition. Then, the right tibiae of all rats were fractured (osteotomized) using pulsed ultrasound (Piezosurgery^®^, Mectron Medical Technology, Carasco, Italy) and fixed with a T-shaped titanium fixation plate XS (57-05140 Stryker Trauma, Selzach, Switzerland) in a fashion that produced an osteotomy gap of 0.5 mm [60]. The animals were injected with 5 mg/kg enrofloxacin (Baytril^®^—Bayer Korea Ltd, Seoul, Republic of Korea) intramuscularly every 12 h for 7 days, and 0.1 mg/kg buprenorphine (0.342 mg/mL) subcutaneously daily for 3 days, to prevent infection and manage postoperative pain.

### 4.3. Study Design

**Figure 5:**
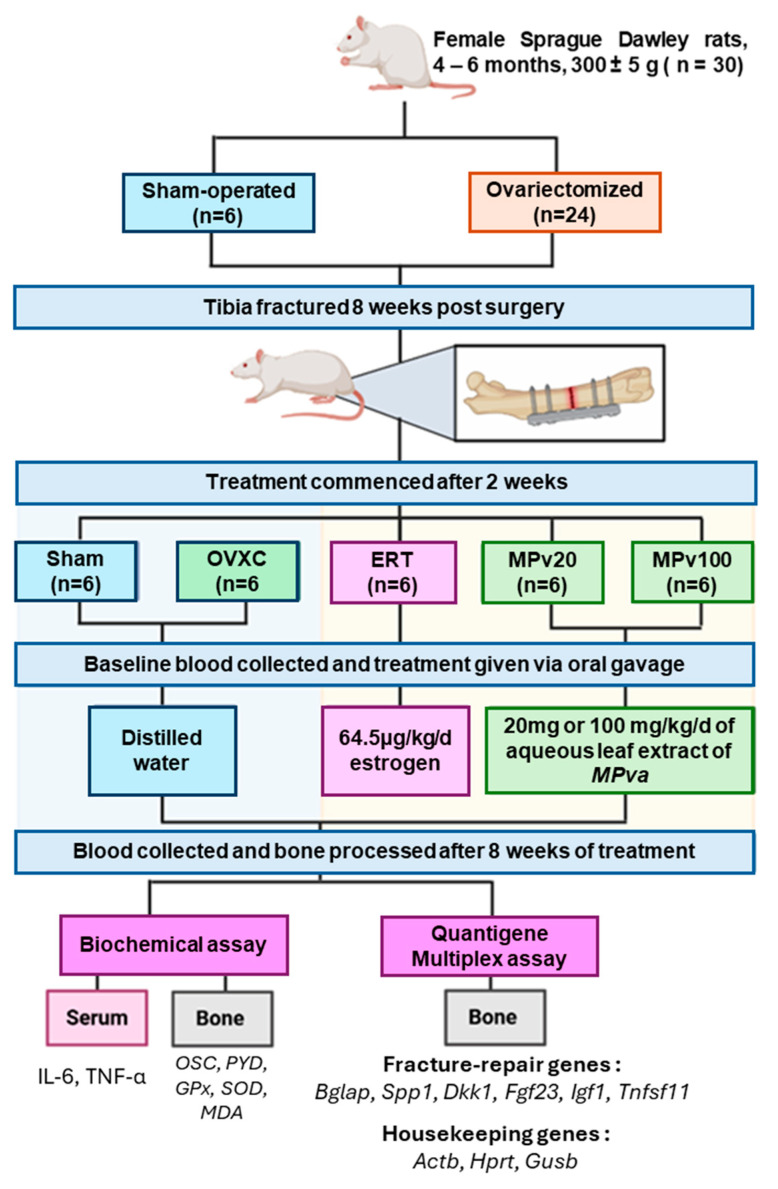
Experimental design. The study involved female Sprague Dawley rats (4–6 months old, 300 ± 5 g, *n* = 30), who were divided into five groups: sham-operated (Sham, *n* = 6); ovariectomized control (OVXC, *n* = 6); ovariectomized rats treated with estrogen (ERT, *n* = 6) at a dose of 64.5 μg/kg/day; ovariectomized rats treated with 20 mg/kg/day of aqueous leaf extract of *M. pva* (MPv20, *n* = 6); and ovariectomized rats treated with 100 mg/kg/day of aqueous leaf extract of *M. pva* (MPv100, *n* = 6). All rats underwent tibia fracture 8 weeks after ovariectomy. Treatments commenced 2 weeks post fracture. Treatments were administered daily via oral gavage for 8 weeks. At the end of the treatment period, blood and bone samples were collected for analysis. Biochemical assays were performed on serum samples to evaluate the levels of IL-6 and TNF-α. Bone samples were analyzed for bone-turnover markers, including osteocalcin (OSC) and pyridinoline (PYD), as well as oxidative stress markers, including glutathione peroxidase (GPx), superoxide dismutase (SOD), and malondialdehyde (MDA). Quantigene multiplex assays were used to assess the expression of fracture-repair genes, including *Bglap*, *Spp1*, *Dkk1*, *Fgf23*, *Igf1*, and *Tnfsf11*, along with housekeeping genes (*Actb*, *Hprt*, and *Gusb*).

Thirty rats were randomly assigned into five groups of six rats each (*n* = 6): a sham-operated group (Sham), an ovariectomized control group (OVXC), an estrogen treatment group (ERT), and two leaf extract treatment groups (MPv20 and MPv100). The sample size of each group was adopted from a previous study [24]. After fracture, the rats had a 2-week healing period before treatment commenced. The ERT group was treated with 64.5 μg/kg/day estrogen (Premarin^®^ Pfizer Ireland Pharmaceuticals, Kildare, Ireland), while the MPv20 and MPv100 groups received 20 mg and 100 mg/kg/day doses of aqueous leaf extract of *MPva* in distilled water, respectively, for 8 weeks. The treatment agents were given orally via oral gavage. The duration of the treatment period was based on a previous study [22]. Blood samples were collected from the rats before and after treatment for the assay of pro-inflammatory cytokines. At the end of the treatment, all rats were humanely sacrificed using an overdose of ketamine-xylazine followed by cervical dislocation, in accordance with AVMA guidelines on euthanasia [61]. The fractured tibiae were excised and pulverized into powder using liquid nitrogen. An appropriate amount of pulverized bone sample was homogenized with an Omni Bead Ruptor and assayed for bone-turnover markers, oxidative markers, and gene expression. The experimental design of the study is shown in Figure 5. The reports of this study comply with the Animal Research: Reporting of In Vivo Experiments (ARRIVE) guidelines for reporting animal research [62].

### 4.4. Blood and Bone Sample Preparation

A 2 mL volume of blood was collected from the tail vein of each rat, before and after treatment, and allowed to stand for 3 h in a 5 mL plain vacutainer tube. After coagulation, the blood samples were centrifuged at 3000 rpm (Hereus Labofuge 400—Thermo Fisher Scientific, Dreieich, Germany) for 10 min. The supernatants were collected with a Pasteur pipette into sterile test tubes and stored at −70 °C. At the end of the treatment, bone samples were collected by humanely euthanizing the rats under an overdose of anesthesia, followed by cervical dislocation. Their right tibiae were excised using a surgical blade (size 11) and scissors. The excised tibiae were then carefully cleared of excess surrounding soft tissues, wrapped in sterile gauze soaked in phosphate-buffered saline, and immediately stored at −70 °C. Prior to experimentation, the titanium fixation plates were carefully removed, and the bone samples were cut, pulverized in liquid nitrogen using a porcelain pestle and mortar, and further disrupted using an Omni Bead Ruptor 24 (Omni International Inc, Kennesaw, GA, USA).

### 4.5. Biochemical Assay

Serum samples from rats were used for biochemical assays, using Enzyme-linked Immunosorbent Assay (ELISA), of the pro-inflammatory cytokines IL-6 and TNF-α, while pulverized bone samples from the right tibiae were used for the bone-turnover markers osteocalcin (OSC) and pyridinoline (PYD), as well as for the oxidative status markers glutathione peroxidase (GPx), superoxide dismutase (SOD), and malondialdehyde (MDA). ELISA kits, sourced from reputable manufacturers through licensed distributors, were employed for these assays. Rat TNF-α and IL-6 kits from Abcam (Waltham, MA, USA) were used to assay TNF-α and IL-6, while rat PYD and OC/BGP kits by Elabscience Biotech (Houston, TX, USA) were used to assay PYD and OSC. The bone oxidative status markers MDA and GPx were assayed with the NWK-MDA and EGPX-100 kits by Northwest Life Science (Vancouver, WA, USA), while SOD was assessed with the ES-OD-100 kit by Bioassay Systems, Hayward, CA, USA. The ELISA protocols for each biochemical marker were conducted following the manufacturer’s instructions provided in the user guide inserts. Absorbance measurements of the samples were performed using an ELISA reader (Thermo Fisher Scientific Sdn. Bhd., Waltham, MA, USA; no. 1510-01170).

### 4.6. QuantiGene Plex Assay

Gene expressions in the powdered bone samples were measured using the QuantiGene 2.0 assay [63]. Six specifically designed probes (Affymetrix, Santa Clara, CA, USA) against genes known to play significant roles in fracture repair—namely, bone gamma-carboxyglutamate (*Bglap*), secreted phosphoprotein 1 (*Spp1*), Dickkopf 1 homolog (*Dkk1*), fibroblast growth factor (*Fgf23*), insulin growth factor (*IgfF1*), *Tnfsf11*, and 3 housekeeping genes (HKGs): β-actin (*Actb*), hypoxanthine phosphoribosyltransferase-1 (*Hprt*), and glucuronidase-β (*Gusb*), representing high, medium, and low expression levels, respectively— were utilized in this study. Ribonucleic acid (RNA) was extracted from 10 mg of powdered bone samples using the QuantiGene Sample Processing Kit, which contains 4 μL of proteinase K (Thermo Fisher Scientific). The resultant RNA underwent target capturing, signal amplification, and RNA target detection using a Luminex Bio-Plex 200 system (Luminex Corp., Austin, TX, USA). Each sample was analyzed in triplicate. The total fluorescence from each individual bead in each well, corresponding to individual mRNA molecules minus the background fluorescence for that bead type, was normalized to the geometric mean of the fluorescence of the housekeeping genes (HKGs). The normalized signals for individual mRNAs from triplicate wells were averaged to yield a single value for each mRNA species, measured and reported as the median fluorescence intensity (MFI). These values were then processed to determine the final output of gene fold changes.

### 4.7. Statistical Methods

Statistical analysis was performed using SPSS version 20. Data were first tested for normality of distribution using the Shapiro–Wilk test. Subsequently, statistical variance was analyzed using one-way ANOVA, followed by Tukey’s post hoc test. Further comparisons between pre- and post-treatment values within the same experimental group were conducted using Student’s *t*-test. A *p*-value of <0.05 was considered statistically significant.

## 5. Conclusions

**Figure 6:**
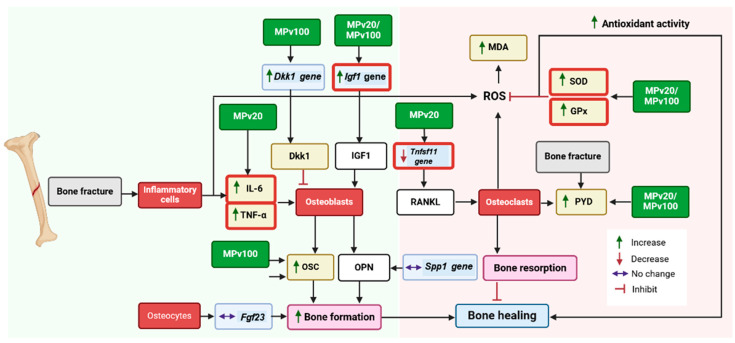
Proposed mechanisms of fracture healing modulated by *MPva* leaf extract.

The fracture-healing potential of *MPva* appears to involve its ability to modulate serum pro-inflammatory cytokines and enhance the bone antioxidant status. These effects are likely mediated through the regulatory influence of *MPva* leaf extract on key genes such as *Igf1* and *Tnfsf11*, which play pivotal roles in bone remodeling processes, as illustrated in Figure 6. These findings align with previous studies that have highlighted the fracture-healing properties of *MPva*, further reinforcing its potential as a complementary or alternative medicine for managing osteoporosis-related fractures, particularly in postmenopausal conditions. Future clinical studies are essential to validate these effects and explore the broader therapeutic potential of *MPva* in bone health management.

This diagram illustrates the pathways involved in bone-fracture healing influenced by *MPva* leaf extract. Following a bone fracture, an inflammatory response is triggered, characterized by elevated levels of pro-inflammatory cytokines (IL-6 and TNF-α) released by neutrophils and macrophages. These cytokines contribute to inflammation and interact with cellular pathways involved in bone remodeling. The *MPva* extract mitigates oxidative stress by boosting the activity of antioxidant enzymes (SOD and GPx). MPva modulates the expression of key genes involved in bone remodeling, including *Tnfsf11* (inhibiting bone resorption) and *Igf1* (promoting bone formation). Enhanced osteoblast activity, evidenced by increased production of osteocalcin (OSC), combined with regulated osteoclast function, facilitates balanced bone turnover. This synergy could contribute to effective bone healing and recovery.

## Figures and Tables

**Table 1:** Effects of *Marantodes pumilum* leaf extract on body weight of fractured osteoporotic rats.

Group	Weekly Animal Weight (g)									
	−1	0	1	2	3	4	5	6	7	8
Sham	240 ± 4	245 ± 5	241 ± 4 *	250 ± 7	259 ± 7 *	257 ± 7 *	253 ± 7 *	255 ± 6 *	257 ± 6 *	256 ± 6 *
OVXC	245 ± 5	266 ± 7	288 ± 9	322 ± 9	333 ± 9	339 ± 11	348 ± 12	351 ± 13	355 ± 13	347 ± 7
ERT	245 ± 4	269 ± 7	294 ± 10	297 ± 10	299 ± 10	306 ± 9	308 ± 10	310 ± 11	316 ± 11	318 ± 11
MPv20	248 ± 2	274 ± 5	306 ± 8	312 ± 10	320 ± 12	328 ± 10	327 ± 11	328 ± 11	336 ± 13	338 ± 13
MPv100	246 ± 4	269 ± 4	290 ± 9	310 ± 10	313 ± 11	322 ± 12	324 ± 14	333 ± 14	335 ± 14	335 ± 13

* Significant difference when compared with other groups (*p* < 0.05). Sham: sham-operated; OVXC: ovariectomized control; ERT: estrogen treatment 64.5 μg/kg/day; MPv20: 20 mg/kg leaf extract of *Marantodes pumilum*; and MPv100: 100 mg/kg leaf extract of *Marantodes pumilum.*

**Table 2:** Antioxidant levels in bones of rats treated with *MPva* leaf extract.

	Sham	OVXC	ERT	MPv20	MPv100
GPx activity (mU/mL)	28 ± 0.4	17 ± 0.28	32 ± 0.37 *	38 ± 0.8 *	34 ± 0.33 *
SOD activity (mU/mL)	2230 ± 70 *	1540 ± 70	1730 ± 100	1950 ± 80 *	2120 ± 50 *
MDA	0.96 ± 0.05	1.32 ± 0.11	0.97 ± 0.01	1.00 ± 0.09	0.95 ± 0.02

Data presented as mean ± SEM (*p* < 0.05, ANOVA). * indicates significant difference compared to OVXC group. Sham: sham-operated; OVXC: ovariectomized control; ERT: estrogen treatment 64.5 μg/kg/day; MPv20: 20 mg/kg leaf extract of *Marantodes pumilum*; and MPv100: 100 mg/kg leaf extract of *Marantodes pumilum.*

**Table 3:** Fold change in bone-repair genes in ovariectomized rats.

Genes	HKGs	Sham		OVXC	ERT		MPv20		MPv100	
*Spp1*	*Actb*	0.8229 *	🡫	1	0.8616		0.7851 *	🡫	0.7704 *	🡫
	*Hprt1*	0.0147 *	🡫	1	0.0183 *	🡫	0.0162 *	🡫	0.0177 *	🡫
	*Gusb*	0.0513 *	🡫	1	0.0636 *	🡫	0.0624 *	🡫	0.0584 *	🡫
*Bglap*	*Actb*	1.0925		1	0.9399 ^a^	🡫	0.9322 ^a^	🡫	0.9115 ^a^	🡫
	*Hprt1*	0.7605 *	🡫	1	0.7661 *	🡫	0.7558 *	🡫	0.8188	
	*Gusb*	1.0012		1	1.0289		1.0914		1.0146	
*Igf1*	*Actb*	1.4358 *	🡩	1	1.0517 ^a^	🡩	1.0881 ^a^	🡩	1.0678 ^a^	🡩
	*Hprt1*	1.0064		1	0.8524 *^,a^	🡫	0.8828		0.9565	
	*Gusb*	1.3135 *	🡩	1	1.1416 ^a^	🡩	1.2832		1.1822	
*Dkk1*	*Actb*	0.9736		1	1.2919		1.2406		1.4449	
	*Hprt1*	0.6923		1	1.0554		1.0119		1.3273 ^a^	🡩
	*Gusb*	0.8825		1	1.3870		1.4503		1.5658 ^a^	🡩
*Tnfsf11*	*Actb*	0.9793		1	0.7701 *^,a^	🡫	0.7885 *^,a^	🡫	0.8353 *	🡫
	*Hprt1*	0.6706 *	🡫	1	0.6215 *	🡫	0.6263 *	🡫	0.7444 *	🡫
	*Gusb*	0.9096		1	0.8479		0.9455		0.9516	
*Fgf23*	*Actb*	1		1	0.7950 *^,a^	🡫	0.8034 *^,a^	🡫	0.7990 *^,a^	🡫
	*Hprt1*	1		1	0.9387		0.9336		1.0331	
	*Gusb*	1		1	0.9428		1.0273		0.9713	

* indicates significant difference compared to OVXC group; ^a^ indicates significant difference compared to Sham group (*p* < 0.05, ANOVA); 🡩🡫 indicate upregulation or downregulation compared to OVXC group. Sham: sham-operated; OVXC: ovariectomized control; ERT: estrogen treatment 64.5 μg/kg/day; MPv20: 20 mg/kg leaf extract of *Marantodes pumilum*; and MPv100: 100 mg/kg leaf extract of *Marantodes pumilum*. Bone gamma-carboxyglutamate (*Bglap*, secreted phosphoprotein 1 (*Spp1*), Dickkopf 1 homolog (*Dkk1*), fibroblast growth factor (*Fgf23*), insulin growth factor (*Igf1*), *Tnfsf11*, and 3 housekeeping genes (*HKGs*): β-actin (*Actb*), hypoxanthine phosphoribosyltransferase-1 (*Hprt*), and glucuronidase-β (*Gusb*).

## Data Availability

The original contributions presented in this study are included in the article. Further inquiries can be directed to the corresponding author.
